# Attention-guided sampling for colorectal cancer analysis with digital pathology

**DOI:** 10.1016/j.jpi.2022.100110

**Published:** 2022-06-24

**Authors:** Andrew Broad, Alexander I. Wright, Marc de Kamps, Darren Treanor

**Affiliations:** a School of Computing, University of Leeds, Sir William Henry Bragg Building, Woodhouse Lane, Leeds LS2 9BW, UK; b Leeds Institute for Data Analytics, University of Leeds, Level 11, Worsley Building, Clarendon Way, Leeds LS2 9NL, UK; c Leeds Teaching Hospitals NHS Trust, Beckett St, Harehills, Leeds LS9 7TF, UK; d Division of Pathology and Data Analytics, Leeds Institute of Medical Research, University of Leeds, St James’s University Hospital, Leeds LS9 7TF, UK; e The Alan Turing Institute, 96 Euston Road, London NW1 2DB, UK; f University of Leeds, Leeds LS2 9JT, UK; g Department of Clinical Pathology, and Department of Clinical and Experimental Medicine, Linköping University, Linköping, Sweden; h Center for Medical Image Science and Visualization (CMIV), Linköping University, Linköping, Sweden

**Keywords:** Artificial intelligence, Attention, Colorectal cancer, Region of interest, Sampling, Tumour–stroma ratio

## Abstract

Improvements to patient care through the development of automated image analysis in pathology are restricted by the small image patch size that can be processed by convolutional neural networks (CNNs), when compared to the whole-slide image (WSI). Tile-by-tile processing across the entire WSI is slow and inefficient. While this may improve with future computing power, the technique remains vulnerable to noise from uninformative image areas.

We propose a novel attention-inspired algorithm that selects image patches from informative parts of the WSI, first using a sparse randomised grid pattern, then iteratively re-sampling at higher density in regions where a CNN classifies patches as *tumour*. Subsequent uniform sampling across the enclosing region of interest (ROI) is used to mitigate sampling bias. Benchmarking tests informed the adoption of VGG19 as the main CNN architecture, with 79% classification accuracy. A further CNN was trained to separate false-positive normal epithelium from tumour epithelium, in a novel adaptation of a two-stage model used in brain imaging.

These subsystems were combined in a processing pipeline to generate spatial distributions of classified patches from unseen WSIs. The ROI was predicted with a mean F1 (Dice) score of 86.6% over 100 evaluation WSIs. Several algorithms for evaluating tumour–stroma ratio (TSR) within the ROI were compared, giving a lowest root mean square (RMS) error of 11.3% relative to pathologists’ annotations, against 13.5% for an equivalent tile-by-tile pipeline. Our pipeline processed WSIs between 3.3x and 6.3x faster than tile-by-tile processing.

We propose our attention-based sampling pipeline as a useful tool for pathology researchers, with the further potential for incorporating additional diagnostic calculations.

## Introduction

The gigapixel resolution of whole-slide images (WSIs) is a source of valuable diagnostic information to pathologists. However, there is a worldwide shortage of pathologists,^1^ exposing a need for tools to ease diagnostic bottlenecks when examining WSIs. Artificial Intelligence (AI) algorithms such as convolutional neural networks (CNNs) can classify tissue types, but these operate on much smaller images.^2^

A key problem in applying AI to digital pathology lies in reconciling this capability with the comparatively vast scale of a WSI. Results obtained in this work for tile-by-tile classification across the entire WSI area showed this approach to be prohibitively slow, and noise from less informative regions led to false-positive ‘tumour’ classifications outside the ROI. This suggests that a viable AI solution must reduce the volume of information from the WSI that is irrelevant to making a contextual diagnosis, but nonetheless reliably detect diagnostically important features such as regions of tumour.

We propose a solution based on attention, a cognitive process in which features of interest are selected from a complex, high-resolution input. In human vision, the small central fovea of the eye gathers information from a small, high-resolution sample of the scene. The brain directs multiple fixations and assembles these into an internal representation of the scene, requiring less bandwidth than processing the whole scene at full resolution.^3^

Attention-like processes have been used to reduce the rate of false positives (FP) in diagnostic imaging, in a manner akin to searching for objects in one’s peripheral vision, before directing high-resolution glimpses to examine the targets more closely. One trial,^4^ studying white matter hyperintensities in migraine patients, used two cascaded convolutional neural networks (CNNs) to detect anomalies in brain magnetic resonance imaging (MRI) scans. The first CNN identified likely disease locations, and was tuned for high sensitivity at the expense of selectivity. These regions were then analysed with higher selectivity by the second CNN stage, reducing the rate of false positives.

In digital pathology, attention-like processes have been used to guide patch-scale AI algorithms towards informative areas of the WSI.^5^, ^6^, ^7^ Cruz-Roa *et al*^8^ presented a Quasi Monte Carlo method for estimating the region of interest (ROI) containing suspected tumour. Their iterative algorithm initially sampled a uniform pattern of randomly offset patches, using a CNN to classify these by cell type. Where adjacent samples yielded different cell classes, additional intermediate samples were then taken. As this process was repeated, the patch sampling was concentrated near transitions between cell regions. This gave an efficient estimation of the ROI boundary, but with less dense sampling across the ROI.

Given an algorithm that can predict the ROI, further diagnostic metrics can be derived from the image patches within this region. In colorectal and other cancers, the proportion of cancer cells in relation to background supportive tissue (stroma)—the so-called tumour–stroma ratio (TSR)—is a significant predictor of survival^9^ or recurrence.^10^ High tumour stroma (>50%) correlates with increased rates of disease recurrence.^11^

The patch size used in WSI analysis influences classification accuracy. There is a trade-off between isolating only the ground-truth sampling point, and incorporating enough surrounding tissue to provide structural context.^12^ Scanning and staining quality are also key to reliable analysis, and an optimal workflow must include a quality control (QC) step to monitor image quality.^13^

Our WSI-processing pipeline represents a novel extension of Cruz-Roa’s algorithm, predicting the ROI whilst enclosing a uniform sampling distribution required for accurate TSR calculation.

## Material and methods

### Data preparation

Our WSI processing pipeline was trained and evaluated using data from the QUASAR study,^14^ which investigated the benefits of adjuvant chemotherapy in colorectal cancer resection surgery, and explored the use of TSR as a predictor of survival.^11^ For this, 2211 slides of haematoxylin and eosin (H&E) stained tissue were scanned with an Aperio XT system (Leica Biosystems, Vista, California, USA) at 0.49 μm/pixel, with JPEG 2000 compression at 49.09 compression ratio and a quality factor of 30.^11^

Pathologists in the QUASAR study annotated each of the resulting 2211 WSIs with the tumour outline (ROI), and tissue classifications for approximately 50 locations in each WSI. The 50 classification locations were allocated within a 3 mm square box, 1 per WSI, placed for maximum density of tumour cells and factors such as proximity to the luminal aspect (interior bowel wall). Within the 3 mm box, the sampling locations were determined using RandomSpot software by Wright,^15^ which uses a hexagonal grid with a random starting point to minimise sampling bias.

**Fig. 1:**
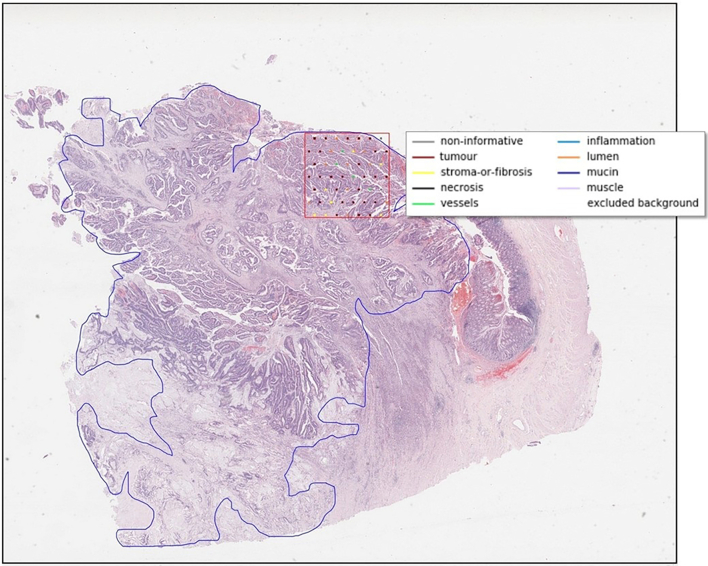
Annotated QUASAR data, showing WSI overlaid with pathologist-generated ROI (blue outline) and cell patch classifications (in 3 mm box, red).^14^

A subset of 689 WSIs, with mean size 175.3 MB, satisfied QC criteria^13^ for slide mounting and scanning quality and were used in our work. Annotation data for ROI and tissue class were supplied in eXtended Markup Language (XML) files, 1 per WSI for each annotation type. A typical QUASAR WSI with expert annotations overlaid is shown in Fig. 1.

**Fig. 2:**
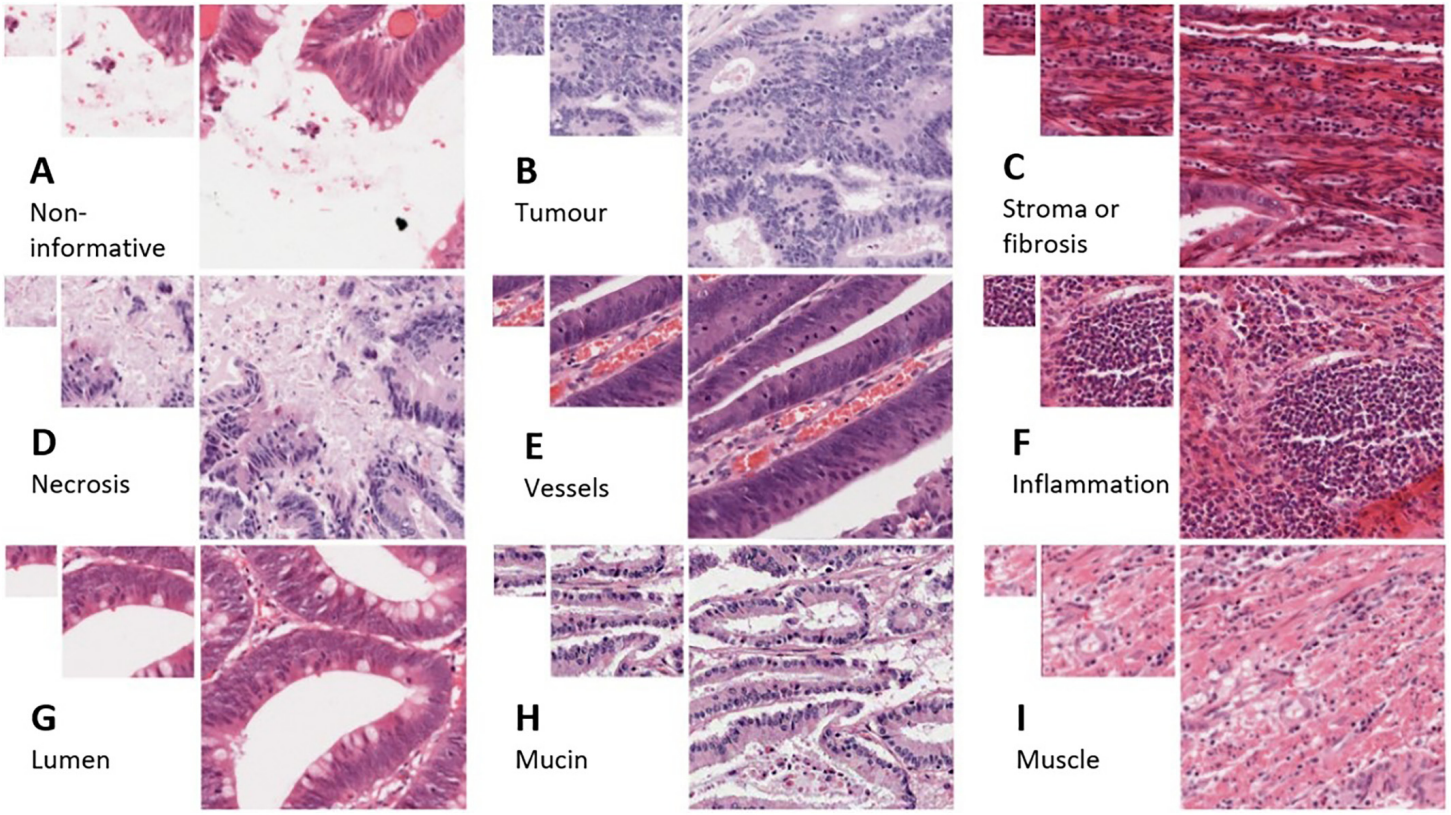
Typical cell patches extracted from QUASAR slides. Tissue types (left-right, top-bottom): A) non-informative, b) tumour, c) stroma or fibrosis, d) necrosis, e) vessels, f) inflammation, g) lumen, h) mucin, and i) muscle. Patch sizes shown: 100x100px, 224x224px, 448x448px. The pathologists’ ground-truth classification refers to the central pixel of each patch, although surrounding tissue was examined to provide structural context.

Fig. 2 shows the 9 image classes used in the ground-truth annotation: *non-informative, tumour, stroma or fibrosis, necrosis, vessels, inflammation, lumen, mucin,* and *muscle.* The non-informative class represents tissue that was unclassifiable as other types, whether this is due to blank background and a lack of surrounding context that would identify the gap as lumen or vessel, or due to a mix of cell types from which a dominant tissue type could not be inferred at that location.

Image patches were extracted from the QC-passed WSIs at locations corresponding to the original RandomSpot sampling points, then saved to sub-directories corresponding to the pathologist’s classification. A patch size of 224×224 pixels was chosen for compatibility with established convolutional neural network (CNN) architectures. This corresponds to a square of side 0.11 mm at the 0.49 μm/pixel QUASAR image resolution. This was found to enclose sufficient structural context around the sample point to enhance classification accuracy, for example when distinguishing lumen from blood vessels or background in images where the central region of the patch is empty.

### Convolutional neural network training and benchmarking

CNNs were trained to classify the QUASAR-derived patches into our 9 tissue classes. Architectures were shortlisted for their performance on general challenge image sets such as ImageNet,^16^ as well as for their popularity in recent pathology AI experiments.^17^^,^^18^ PyTorch^19^ CNN implementations of VGG16, VGG19, AlexNet, DenseNet, GoogLeNet, Inception, MobileNet, and ResNet, were trained and tested for overall classification accuracy using the patches. Where available, models pre-trained on ImageNet were also used. Confusion matrices were then examined for systematic misclassification, especially between tumour and stroma.

The randomly initialised CNNs were trained for 100 epochs to ensure full convergence. ImageNet-pre-trained versions were trained for 25 epochs. The CNNs were implemented in Python v3.7 with PyTorch v1.2.0 and CUDA v10.0.130. We used Stochastic Gradient Descent (SGD) with a learning rate of 0.001 and momentum of 0.9. Training, test, and evaluation patch sets were created by randomly grouping the 689 parent WSIs in the ratio 489:100:100. The training process was executed on NVIDIA V100 Graphics Processing Units (GPUs) on the Advanced Research Computing 4 (ARC4) system at the University of Leeds.

The best-performing CNN architectures from these experiments were then selected for use in the image processing pipeline. Here, the resulting percentage of tumour-classified points within the original ground-truth ROI was logged as a further indicator of CNN performance.

### CNN for false-positive detection

In the QUASAR annotated data, all patches were sampled from within the annotated ROI. We believe this may increase the risk of bias in a classifier trained on this data alone, particularly when classifying patches from other regions of the WSI. QUASAR patches annotated as ‘tumour’ actually represent *tumour epithelial* cells. Initial experiments showed that patches of normal epithelium, from outside the ROI, were being classified as tumour. Sporadic patches of false-positive tumour had previously led to inaccurate predictions of the tumour ROI outline, with the attendant risk of misdiagnosis.

A two-way VGG16 CNN classifier was trained to distinguish between normal and tumour epithelium. The training data consisted of two balanced directories, each with 28,589 patch images labelled as tumour and normal epithelium respectively. These were identified by applying the original CNN classifier to patches sampled from throughout the WSI. Patches classified as ‘tumour’ were assumed to contain epithelial tissue. The expert-annotated ROIs allowed us to sort these patches into ‘tumour epithelium’ (inside ROI) and ‘normal epithelium’ (outside ROI), for use in training the two-way classifier.

### WSI analysis pipeline

**Fig. 3:**
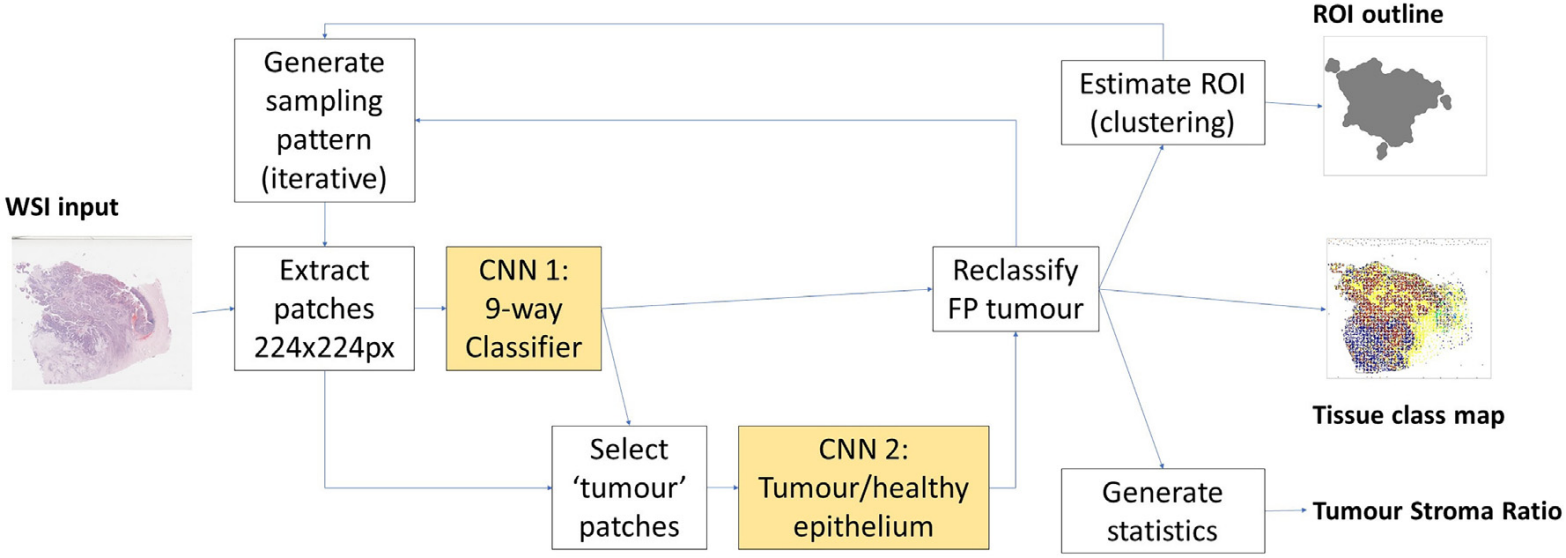
WSI sampling and patch-classification pipeline.

The complete pipeline is shown in Fig. 3. The first processing cycle used a sparse, quasi-random sampling pattern. The WSI area was divided into a coarse square grid, using spacings of 640, 768 and 1024px, corresponding to 313 μm, 376 μm and 502 μm at 0.49 μm/pixel resolution. A 224px (110 μm) square region was allocated randomly in each grid box. The WSI was sampled at these locations and each resulting patch was classified using the 9-class CNN.

Patches predicted to be of class ‘tumour’ were then passed to the second CNN, to further categorise these patches as ‘true positive’ tumour epithelium, or ‘false positive’ normal epithelial cells. This process was inspired by Hong’s two-stage FP-correction^4^ in MRI processing.

A second iteration of sampling patch generation was then performed. Wherever a tumour patch was reported, a further 4 sampling patches would be placed randomly within the tumour patch’s parent grid box. Where the new patch overlapped the existing patch, a new random location would be tried until a clear space was found. Additional image patch data would be extracted from the WSI at the new locations. These new patches were also classified and added to the output class map.

The FP-corrected map of tumour patches and their locations was then used to predict the tumour ROI. Density-Based Spatial Clustering of Applications with Noise (DBSCAN)^20^ was applied to the tumour points, to generate an outline around the regions of tumour points. This algorithm rejected sporadic ‘noise’ points without multiple neighbours in a 2000 pixel radius, so that these would not contribute to the final estimate of the ROI.

A final stage of patch generation and classification was then performed, to obtain a uniformly higher sampling density across the entire ROI. The convex hull of the ROI polygon was evaluated, then new patches were added within this region, up to a limit of 5 patches per grid box within the ROI. New patches would be sampled from the WSI and classified as before, to provide an output of patch locations and classes at a uniform higher density within tumour regions. Non-tumour regions, outside the predicted ROI, would retain the lower original density of one patch per grid box.

To avoid overfitting, the pipeline was assessed using the previously unseen 100 WSIs in the evaluation set, from the data split that provided the test and training patches for CNN training.

### Tile-by-tile reference pipeline

To assess the comparative performance of our sampling pipeline, we developed an additional pipeline to perform patch classification and generate TSR and ROI estimates for WSIs divided into contiguous, tiled image patches. The pipeline was similar to that in Fig. 3 but used a dense tiled sampling pattern in a single processing iteration, to process all areas of the WSI systematically.

### TSR estimation

Now possessing a collection of output patches with tissue class and position data, we explored several algorithms for calculating tumour–stroma ratio. For each WSI in the evaluation set, we estimated the TSR from the proportions of patches classified as *tumour* (T) and *stroma or fibrosis* (S)*,* using the formula:

$$\mathrm{TS}R_{\mathrm{est}}=\frac{T}{T+S}$$

The following sampling algorithms were used in the pipeline, to compare the effects on TSR of different patch sampling regimes:

1) 100 patches uniformly distributed within predicted ROI.
2) 120 patches within 3mm square box (6122x6122px at 0.49 μm/px), using RandomSpot-based triangular grid centred on point of highest tumour patch density, to simulate sampling approach used in QUASAR study.
3) Mean of TSRs based on all sampled patches, within sliding 3 mm box, across predicted ROI.
4) Ground-truth (GT) sampling locations based on original QUASAR data points.
5) 4-layer, 9-channel CNN trained to estimate TSR from patch class totals per parent grid box.

The GT sampling locations were included to assess the maximum theoretical accuracy of our TSR prediction, that could be achieved if it were possible to predict the exact sampling location that a pathologist would choose for TSR calculations. It is acknowledged that this approach would not be available for unseen WSIs.

The ground-truth *TSR*_*GT*_ was derived from the totals of RandomSpot samples that the original pathologist had classified as *tumour* (T) and *stroma or fibrosis* (S) for each WSI*.* The accuracy of the TSR predicted by our pipeline was calculated for each algorithm under test, using root mean square error (RMSE), calculated across the *N* WSIs in the evaluation set according to:

$$\mathrm{RMSE}=\sqrt{\frac{1}{N}\sum_{i=1}^{N}{\left(\mathrm{TS}R_{GT_{i}}-\mathrm{TS}{R_{\mathrm{est}}}_{i}\right)}^{2}}$$

The mean error (ME) across all evaluation WSIs was also recorded for each method of calculation. The ME is the mean difference between ground truth and estimated values:

$$\mathrm{ME}=\frac{1}{N}\sum_{i=1}^{N}\left(\mathrm{TS}R_{GT_{i}}-\mathrm{TS}R_{\mathrm{es}t_{i}}\right)$$

The pipeline was executed for grid boxes of 640, 768, and 1024px on each side, and with both one and two iterations of re-sampling before the final whole-ROI sampling step. ROI estimates were compared with the ground-truth ROI using F1 (Dice) score. TSR mean and RMS errors were logged for each of the sampling methods under test.

## Results

### CNN benchmarking

Table 1 shows the classification accuracies (percentage of correct classifications), model size and inference time for the CNN architectures we assessed.

**Table 1:** Comparative performance of CNN architectures in 9-way classification of 224x224px QUASAR patches.

CNN type	Model size (MB)	Inference time (ms/patch)	Accuracy (random initial weights)	Accuracy (pre-trained on ImageNet)	Best % tumour in ROI from pipeline (pre- FP correction)
VGG19	548	63.9	72%	**79%**	**94.8%**
GoogLeNet	25.4	63.8	**79%**	75%	92.0%
DenseNet	111	35.6	74%	78%	93.7%
VGG16	528	35.3	74%	78%	91.6%
MobileNet	13.6	18.2	73%	77%	91.0%
AlexNet	233	18.9	75%	76%	
Inception 3	91.2	57.8	72%	70%	
ResNet 50	97.8	35.0	71%	Model unavailable	
ResNet 18	44.7	37.1	68%	68%	

The highest percentage score in each column is shown in bold text.

The VGG19 gave the joint-best accuracy, and the highest percentage of predicted tumour points inside the ground-truth ROI. This model was therefore selected as the main classifier (CNN1 in Fig. 3) for subsequent pipeline experiments.

### False-positive correction

The pre-trained VGG16 model, trained to distinguish false-positive normal epithelium from true-positive tumour epithelium, recorded an overall accuracy of 92.7% against the test set. Measuring before the FP-correction stage, 91.6% of predicted tumour was within the annotated ROI. This value increased to 97.1% when measured after false positive correction.

### Classification plots

**Fig. 4:**
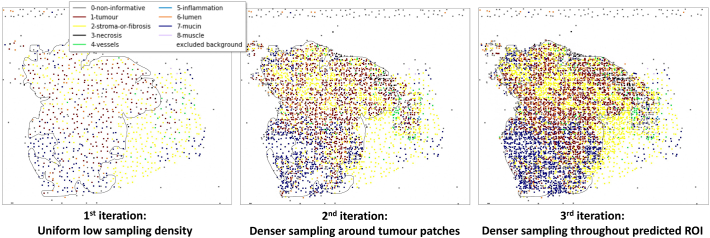
Sampling iterations applied during WSI processing, showing increasing density around detected tumour patches.

Fig. 4 shows the output classification plots for QUASAR WSI *42020.svs* after each processing step, with the ground-truth ROI outline for reference. The first iteration shows the initial uniform sampling pattern (with background areas excluded). After resampling around predicted tumour locations, the denser sampling pattern begins to emerge in this region. The final iteration reveals a uniform, random distribution of sample patches across the estimated region of interest.

### ROI estimation

The output polygons for the estimated ROI were compared with the pathologist’s original ROI annotations in the QUASAR dataset, by calculating the mean Dice Similarity Coefficient (DSC, or F1 score) for all WSIs in the evaluation set.

Table 2 shows the F1 score obtained using 1 and 2 sampling steps, between the initial sparse iteration and the final whole-ROI dense sampling stage, for the 3 different parent grid box sizes. Pipeline performance is also given in terms of per-image processing time and mean total patches processed per WSI.

**Table 2:** Pipeline performance and ROI accuracy for various sampling grid sizes and numbers of sampling iterations. The 224px grid size represents tile-by-tile processing, for comparison.

Grid h, w (pixels)	Grid h, w (μm)	Resampling iterations (before final step)	Processing time, mins per WSI	Patches per WSI	ROI agreement (F1 Score)
1024	502	1	23:19	4041	79.3%
1024	502	2	23:05	7007	83.0%
768	376	1	25:12	6542	83.2%
768	376	2	36:27	12,016	86.5%
640	313	1	29:05	7826	83.6%
640	313	2	44:18	14,257	86.6%
224	110	–	145:21	33,065	89.3%

Table 3 shows the lowest RMS error in the TSR calculation, for each combination of grid size and resampling iterations. In both Table 2 and Table 3, the 224px grid spacing equates to systematic tile-by-tile processing at our 224x224px patch size.

**Table 3:** Accuracy of tumour–stroma ratio estimation for various sampling grid sizes and numbers of sampling iterations. The 224px grid size represents tile-by-tile processing, for comparison.

Grid h, w (pixels)	Resampling iterations (before final step)	Min TSR RMSE	Sampling strategy giving min RMS error
1024	1	12.7%	100 patches, estimated ROI
1024	2	12.9%	3 mm box, max tumour density point
768	1	12.7%	100 patches, estimated ROI
768	2	11.8%	3 mm box, max tumour density point
640	1	11.3%	3 mm box, max tumour density point
640	2	12.4%	Mean TSR over estimated ROI.
224	-	13.5%	3 mm box, max tumour density point

### Tumour–stroma ratio

A breakdown of the comparative performance of all TSR algorithms is shown in Table 4. These are given for the pipeline configuration that delivered the lowest RMS TSR error, i.e., one resampling iteration at 640px grid size.

**Table 4:** Comparative performance of TSR algorithms for 640px grid size, 1 resampling iteration

Sampling region (using post-FPC tumour points unless stated)	TSR mean error (offset)	TSR RMS error
1) Predicted ROI (average over ≈100 points)	0.09	14.9%
2) 3 mm box at max tumour density point with RandomSpot layout (120 points)	-0.04	11.3%
3) Predicted ROI (mean Sliding Window output)	0.05	12.7%
4) Ground-truth locations	0.00	6.4%
5) 4-layer CNN trained for TSR prediction	0.12	20.7%

## Discussion

### CNN benchmarking

Of the CNN models tested, the ImageNet-pretrained VGG19 gave the joint-best classification accuracy (79%) alongside the non-pretrained GoogLeNet. Examination of the confusion matrices revealed that the VGG19 gave the highest rate of correct classifications of tumour patches, explaining this model’s higher proportion of predicted tumour inside the ground-truth ROI. However, the estimated inference time was relatively slow (65.9 ms/patch), and a faster model such as DenseNet (35.6 ms/patch) is therefore recommended for pipeline configurations involving larger patch counts.

### False-positive detection

Use of the CNN trained to distinguish tumour epithelium from normal epithelium resulted in a 2.8-fold reduction in predicted tumour patches outside the ground-truth ROI, enabling a more accurate estimation of this region.

### ROI estimation

The F1-scores in Table 2 show a strong agreement between the predicted and actual ROI over the evaluation set. This increases with decreasing grid box size, corresponding to increasing sampling density. An additional iteration of resampling around tumour patches also gives an improved F1 score, likely to be due to the higher sampling density around the edges of the tumour regions.

The DBSCAN clustering for ROI estimation excludes single tumour points outside the tumour region, by design. These points are rejected as noise caused by occasional classification errors. Tumour patches on an irregular ROI border would nonetheless be included in the ROI outline by DBSCAN, due to the proximity of similar neighbouring patches in the tumour body.

Generally, increasing the number of sampled patches resulted in more accurate ROI prediction, at the expense of a longer processing time. The highest F1 score was seen in tile-by-tile sampling, while the attention-based pipeline in its various configurations was between 3.3 and 6.3 times faster per WSI.

### TSR estimation

As expected, the lowest RMS error (6.4%) in estimated TSR was observed when sampling at the same ground-truth points as the original pathologist. This error is assumed to be due to CNN classification errors, but is lower than the mean classification error for a single image patch. We suggest that averaging across multiple patches reduces the effect of individual misclassifications in the CNN.

For unseen WSIs, the lowest RMS error (11.3%) in estimated TSR was measured when using the smallest grid size, giving a higher sampling density. However, the benefit of increasing density was not as dramatic as that seen with the ROI F1 score, with the RMSE rising again to 13.5% in the extreme case of tile-by-tile processing.

Sampling algorithms that attended to region of densest tumour yielded slightly more accurate TSR values than algorithms that averaged across the whole predicted ROI. In the original QUASAR dataset, the ground-truth classifications were usually evaluated around the maximum perceived tumour density. Emulating this approach gave an optimal TSR prediction as we anticipated.

The TSR-predicting CNN was developed to avoid hard-coding the assumed behaviour of a pathologist, when choosing patch-sampling locations. It was intended that the CNN would learn the optimum spatial weightings to apply to the input patch distribution, when predicting an overall ratio for each WSI. Although we have not yet reached ‘CNN supremacy’ over other methods for calculating TSR, there is much scope for exploring more sophisticated CNN architectures in pursuit of this goal.

### Limitations

Our pipeline has been optimised for mapping H&E stained WSIs. It has not been tested on immunohistochemistry (IHC) staining, which would require re-training on annotated IHC images to process the different staining colours. It is possible that for more general segmentation/characterisation, we would observe higher accuracy with IHC than H&E, because of the greater colour contrast between blue and brown image features, but further work is needed to explore this.

The QUASAR dataset was chosen for its detailed level of annotation for ROI and tissue classifications, representing many hours of pathologists’ effort. The low rate of WSIs passing QC reflects a non-routine research workflow, where some older slides with inconsistent or faded staining were retrospectively scanned for the trial. In a clinical workflow, with more consistent staining and faster transfer from staining to scanning, we anticipate a higher rate of QC-passed WSIs.

The WSIs were captured using Aperio XT scanners as the clinical trial was performed some time ago. While scanning was not replicated on newer instruments, the general principles would be the same and we expect that the improvements in image quality of newer WSIs could further improve the performance of our pipeline.

The original ground-truth class annotations referred to single-pixel points in the WSIs. We extracted 224×224px patches around each point, to allow the CNN to analyse the structural context of, for example, blank background within hollow structures such as vessels. We acknowledge that the enclosing patch area may contain cell classes other than the ground-truth label. We hypothesise that our CNN embeds a spatial bias towards the centre of the image patch, mitigating the misleading effects of surrounding tissue types. Ongoing work is exploring this effect, together with the use of feedback attention to improve classification accuracy for mixed-class patches.

We present our pipeline as a proof of concept, acknowledging that clinical adoption would depend on further development and validation, to meet FDA (US) or CE-mark (Europe) standards. This would include training with new data from a range of state-of-the-art scanners, and testing CNNs and pipeline against further unseen images. Subject to approval, our pipeline might have application in rapid triage of cancer patients according to their TSR and inferred risk of more aggressive disease.

### Conclusions

Overall, we believe that our WSI-processing pipeline represents a useful analytic tool, with the potential to automate labour-intensive assessments of histopathological images. We have showed that this can be achieved using an attention-based pipeline to deliver processing times 3.3 to 6.3x faster than tile-by-tile analysis, with minimal cost in ROI accuracy and improvements in TSR estimation.

## Declaration of interests

The authors declare the following financial interests/personal relationships which may be considered as potential competing interests: Andrew Broad reports financial support was provided by Roche Tissue Diagnostics. Andrew Broad reports financial support was provided by UK Research and Innovation.
